# A Seabed Real-Time Sensing System for In-Situ Long-Term Multi-Parameter Observation Applications

**DOI:** 10.3390/s19051255

**Published:** 2019-03-12

**Authors:** Lanjun Liu, Zhibo Liao, Caiyi Chen, Jialin Chen, Jiong Niu, Yonggang Jia, Xiujun Guo, Zhaowei Chen, Li Deng, Haibo Xu, Tao Liu

**Affiliations:** 1 College of Engineering, Ocean University of China, Qingdao 266100, China; liaozhibo@stu.ouc.edu.cn (Z.L.); chencaiyi@stu.ouc.edu.cn (C.C.); oucjialin@ouc.edu.cn (J.C.); chenzhaowei@stu.ouc.edu.cn (Z.C.); dengli@stu.ouc.edu.cn (L.D.); 2 Key Laboratory of Marine Environment and Geological Engineering Shandong Province, Ocean University of China, Qingdao 266100, China; yonggang@ouc.edu.cn (Y.J.); guojunqd@ouc.edu.cn (X.G.); xuwangri@163.com (H.X.); ltmilan@ouc.edu.cn (T.L.); 3 College of Environmental Science and Engineering, Ocean University of China, Qingdao 266100, China; 4 College of Chemistry and Chemical Engineering, Ocean University of China, Qingdao 266100, China

**Keywords:** seabed observation, buoy, real-time, underwater acoustic communication, satellite communication, remote monitoring, sensing

## Abstract

Aiming at the real-time observation requirements in marine science and ocean engineering, based on underwater acoustic communication and satellite communication technology, a seabed real-time sensing system for in-situ long-term multi-parameter observation applications (SRSS/ILMO) is proposed. It consists of a seabed observation system, a sea surface relay transmission buoy, and a remote monitoring system. The system communication link is implemented by underwater acoustic communication and satellite communication. The seabed observation system adopts the “ARM + FPGA” architecture to meet the low power consumption, scalability, and versatility design requirements. As a long-term unattended system, a two-stage anti-crash mechanism, an automatic system fault isolation design, dual-medium data storage, and improved Modbus protocol are adopted to meet the system reliability requirements. Through the remote monitoring system, users can configure the system working mode, sensor parameters and acquire observation data on demand. The seabed observation system can realize the observation of different fields by carrying different sensors such as those based on marine engineering geology, chemistry, biology, and environment. Carrying resistivity and pore pressure sensors, the SRSS/ILMO powered by seawater batteries was used for a seabed engineering geology observation. The preliminary test results based on harbor environment show the effectiveness of the developed system.

## 1. Introduction

The deep seabed is closest to the Earth’s interior and is an important area for ocean observation [1]. In deep-sea bottom observation, the construction of a seabed real-time observation system is both a new stage and a need of marine scientific research and marine engineering applications. So far, the ocean observation system has developed into a three-dimensional observation network, including satellite remote sensing, land-based ocean observation stations, sea surface buoy arrays, scientific research vessels, submarine buoy arrays, underwater profile buoys, sea bottom observation networks, etc. It can provide basic information and data services in real-time or quasi-real-time in the world [2,3].

The prototype of the sea bed observation system can be traced back to the US Navy’s acoustic monitoring system [4,5]. At present, the sea bed observation system mainly includes self-contained submarine observation instruments, ship-borne real-time observation instruments, and sea bottom observation networks. The self-contained submarine observation instrument can realize long-term in-situ observation, and its observation data is processed and analyzed after the instrument is recovered. It has been widely used in ocean long-term in-situ observation applications. For example, the French NKE company and the Korean Aeronautical University developed a sea bottom self-contained acoustic wave measurement instrument, which is fixed on a seabed support structure and measures the elevation of the sea bottom by acoustic wave propagation time [6,7]. The University of Washington developed a self-contained multi-parameter seabed observation instrument observing the ocean current velocity and the hazy layer [8]. However, self-contained submarine observation instruments cannot meet the needs of real-time or quasi-real-time observation in marine science or engineering applications. The ship-borne real-time observation instrument can observe ocean parameters in real time [9,10]. An example is ROSON, developed by Vandenberg in the Netherlands, which is used to collect soil mechanical parameters of seabed sediments [11]. However, the ship-borne real-time observation and measurement period of the marine parameters is short, and it is impossible to get the long-term dynamic changes of the marine parameters.

Sea bottom observation networks can meet the needs of long-term in-situ real-time observation. The construction of sea bottom observation networks has become the consensus of major marine countries. In 2009, the world’s first large-scale sea bottom observation network, the North East Pacific Time Integrated Undersea Networked Experiment in Canada (NEPTUNE-Canada), was built and realized long-term multi-parameter observation on the seabed [12]. In 2015, Japan completed the Dense Ocean-floor Network system for Earthquakes and Tsunamis (DONET) installation, which was mainly used for earthquake and tsunami observation [13,14]. In 2016, a larger submarine observation system designed by the United States, the Ocean Observatories Initiative (OOI), was officially launched, and all-weather and long-term observation of specific sea areas can be realized through the internet [15]. The European Union’s European submarine observation program set up the European Seas Observatory Network (ESONET) for the Atlantic, Arctic Ocean, Black Sea and Mediterranean Sea observation [16]. ESONET was developed to EMSO (European Multidisciplinary Sea-floor and water column Observatory) for sea bed observation reliability and cost reduction [17]. As for China, in 2006, the key technology project of the submarine observation network test node was launched. In 2009, the construction of the Xiaoqushan experimental station for the East China Sea submarine observation was completed. In 2011, Tongji University and other institutions formally connected the self-developed cable-based seabed observation network with the MARS deep-sea observation network of the United States. With a depth of nearly 900 m and a continuous observation time of more than six months, the data of seafloor chemical substances such as chlorophyll were obtained successfully [18]. In 2013, the Sanya submarine observation network system was put into use, building a solid foundation for the larger seabed observation network construction of China. However, the above-mentioned submarine observation networks are built on a wired network such as optical fiber, which can meet the long-term real-time observation requirements, but has limitations such as deployment difficulty, high deployment cost, and low degree of freedom in the sea area.

For the problems existing in the above-mentioned seabed observation system, it is necessary to develop a seabed long-term in-situ real-time observation system with easy deployment, low deployment cost, and high freedom degree difficulty. The rapid development of satellite communication and underwater acoustic communication technology provides the possibility for building a seabed real-time observation system based on wireless communication. In this paper, aiming at long-term in-situ real-time observation of seabed engineering geology, a seabed real-time sensing system for in-situ long-term multi-parameter observation applications (SRSS/ILMO) is proposed and developed. The proposed SRSS/ILMO can also realize the observation of different fields by carrying different sensors such as those based on marine chemistry, biology, and environment.

## 2. System Design of SRSS/ILMO

### 2.1. System Structure Design

**Figure 1:**
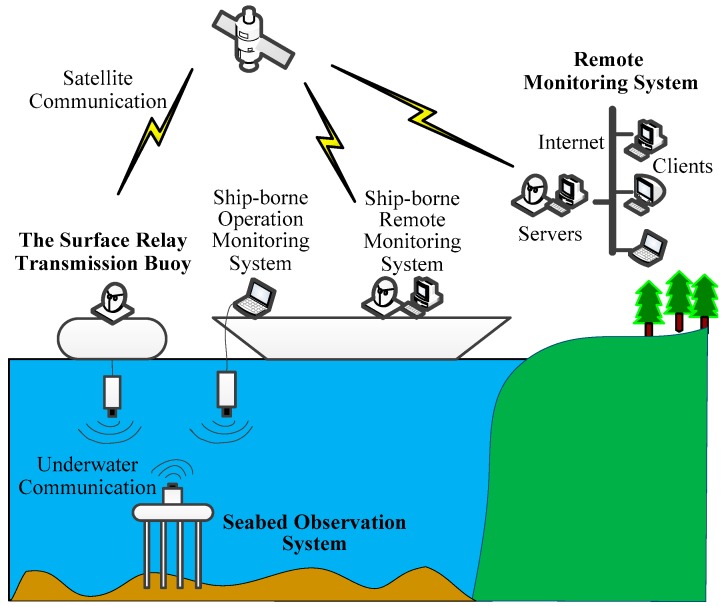
System structure of the seabed real-time sensing system for in-situ long-term multi-parameter observation applications (SRSS/ILMO).

As shown in Figure 1, the proposed SRSS/ILMO system consists of a seabed observation system, a sea surface relay transmission buoy, and a remote monitoring system. The seabed observation system and the sea surface relay transmission buoy exchange information through underwater acoustic communication, and the sea surface relay transmission buoy is connected to the remote monitoring system through satellite communication. At the same time, the seabed observation system can also be directly connected to the ship-borne operation monitoring system through underwater acoustic communication.

**Figure 2:**
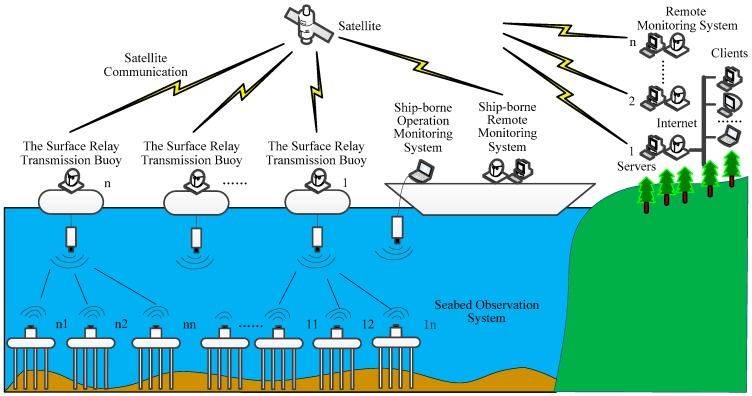
Networked system structure of SRSS/ILMO.

The SRSS/ILMO can also be used for networked observation in the future. As shown in Figure 2, the SRSS/ILMO can support a networked submarine observation system with multiple seabed observation systems, multiple surface relay transmission buoys, multiple remote monitoring systems, and their combination.

### 2.2. System Unit Composition of SRSS/ILMO

**Figure 3:**
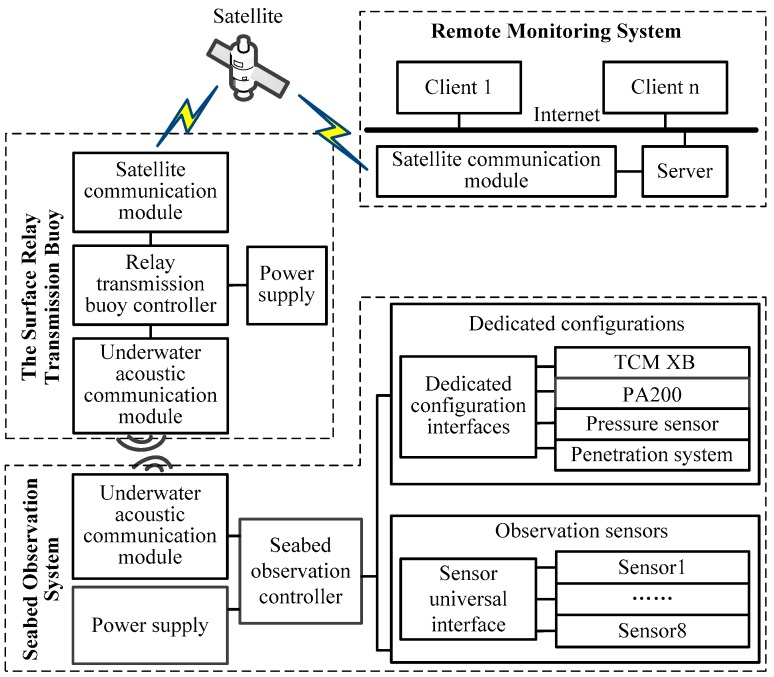
System unit composition of SRSS/ILMO.

The specific system unit composition of SRSS/ILMO is shown in Figure 3. The seabed observation system module includes a seabed observation controller, dedicated configurations, observation sensor interfaces, an underwater acoustic communication module and power supply. The dedicated configurations, including attitude sensor TCM XB, altimeter PA200, pressure sensor, and penetration system, are mainly used for system layout, recovery and attitude monitoring. The observation sensor universal interfaces with universal data interface and conventional power interface are used to carry different observation sensors. The surface relay transmission buoy consists of relay transmission buoy controller, underwater acoustic communication module, satellite communication module, and power supply. The remote monitoring system is an interconnected LAN with clients and servers. 

**Figure 4:**
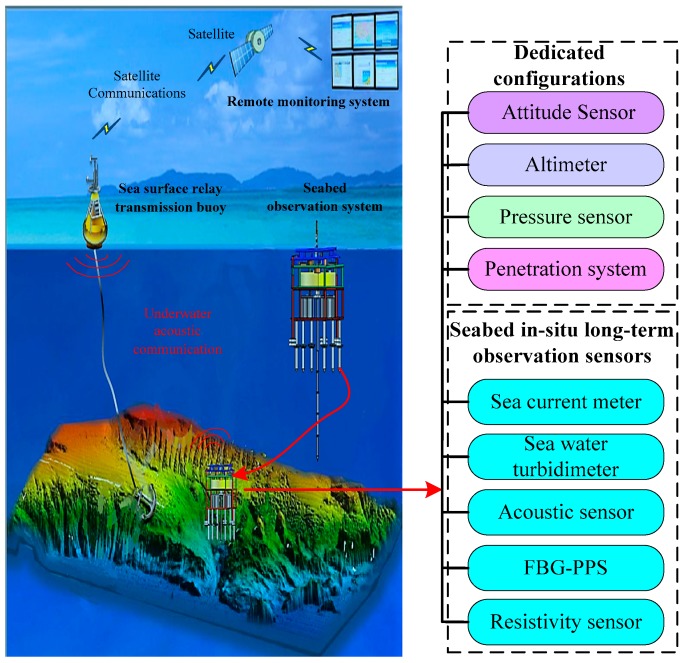
Seabed engineering geology observation of SRSS/ILMO.

As shown in Figure 4, the proposed SRSS/ILMO was used in the project of seabed engineering geology in-situ long-term real-time surveying. The project aims to discover the dynamic change process of the seabed engineering geological environment and its influencing factors by establishing an automatic long-term in-situ continuous observation system. In this project, the seabed observation controller is equipped with the seabed in-situ long-term observation sensors, dedicated configurations, and a seawater battery. Dedicated configurations include the penetration system for placing sensor rods, the pressure sensor used to measure water depth, the altimeter PA200 for measuring altitude off the sea floor, and the attitude sensor TCM XB for attitude measurement of the submarine observing platform. The seabed in-situ long-term observation sensors include a three-dimensional high-density resistivity measurement sensor (referred to as resistivity sensor), a fiber bragg grating based excess pore pressure observation sensor (referred to as FBG-PPS), an acoustic sensor, a sea water turbidimeter, and a sea current meter.

### 2.3. Seabed Observation Controller Design

**Figure 5:**
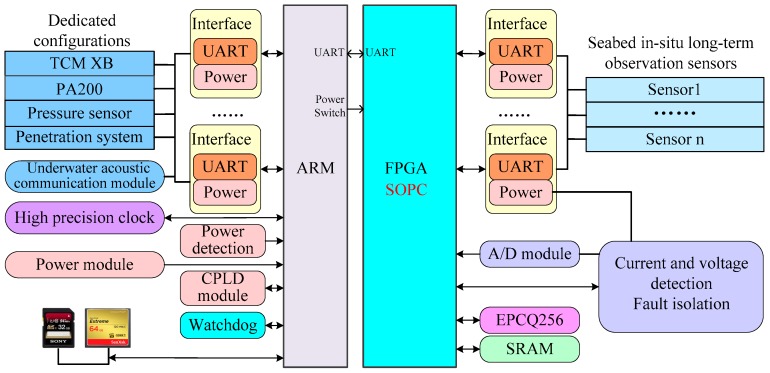
The seabed observation controller.

The seabed observation controller adopts dual microprocessor architecture with an ARM processor and FPGA supporting SOPC (system on a programmable chip) technology. Considering the different working processes, such as placement process, seabed penetration process, seabed observation process, seabed pull up process and recovery process, sensors are assigned to ARM and FPGA by category. ARM connects with the underwater acoustic communication module, PA200, TCM XB, the pressure sensor, and the penetration system needed in the placement and recovery process, seabed penetration and pull up process. At the same time, ARM is responsible for system scheduling, data storage, and low power management. In order to ensure data security, the system uses a dual media card to store data. Seabed in-situ long-term observation sensors are connected to FPGA, which is responsible for observation data collection, current and voltage management, and detection of each sensor. The system uses a star network topology structure to connect various observation sensors. The block diagram of the seabed observation controller is shown in Figure 5.

### 2.4. Relay Transmission Buoy Controller Design

**Figure 6:**
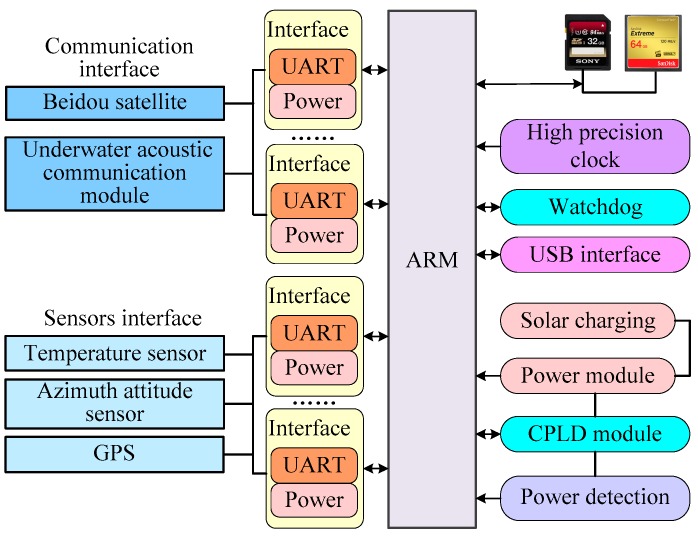
The relay transmission buoy controller.

The relay transmission buoy controller adopts the ARM processor as the main processor to schedule system tasks. The underwater acoustic communication module is used to interact with the seabed observation system. The Beidou satellite is used to communicate with the remote monitoring system. The buoy system is equipped with azimuth attitude sensor TCM XB and GPS for system safety monitoring. In terms of buoy power supply, the combination scheme of solar battery plus lithium battery is adopted. Under the condition of sufficient sunlight for five hours, the energy storage of the lithium battery can meet the power supply demand of the relay transmission buoy system without sunlight for one month. The design of the relay transmission buoy controller is shown in Figure 6.

### 2.5. Remote Monitoring System Design

**Figure 7:**
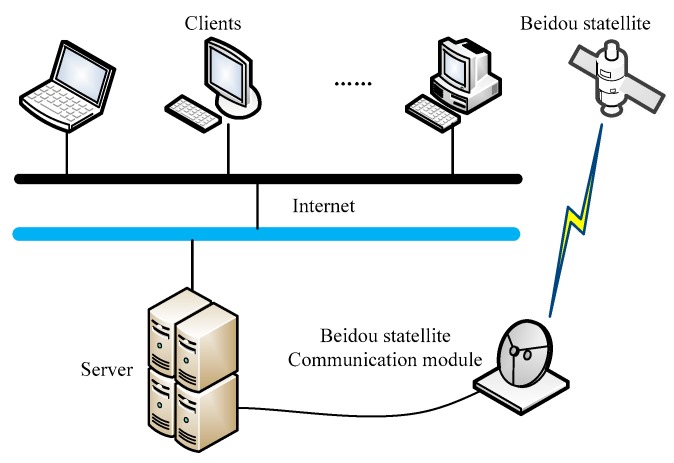
Remote monitoring system.

The remote monitoring system is the monitoring center of SRSS/ILMO, which is composed of the monitoring client software system, the front-end server software system, and the database system. The system composition is shown in Figure 7. The communication function between the monitoring client software and the front-end server software is realized through the internet. The communication between the front-end server software and the Beidou satellite module is standard UART serial communication. The remote monitoring system provides the user’s operation interface to realize the transmission of the control command and working parameters, the recovery of the observation data, and working status information.

The data storage system adopts a distributed design, including the two-level structure of the central server and the observation equipment server. It realizes system information management, data storage, and database backup maintenance of the monitoring software, and enhances the security of data storage and the efficiency of the query operation.

### 2.6. Seabed Observation Flow of SRSS/ILMO

The seabed observation flow of SRSS/ILMO includes the placement process, seabed penetration process, seabed observation process, seabed pull up process, and recovery process. The detailed description of each work process is as follows.

The seabed observation system placement process: The FPGA of the seabed observation system controller and the observation sensors connected to FPGA do not work. The ARM processor works and sleeps periodically. The underwater acoustic communication module is continuously powered, and the seabed observation system communicates with the ship-borne operation monitoring system periodically. The altimeter, pressure sensor, and azimuth attitude sensor connected with the ARM processor collect data periodically.

Seabed penetration process: The FPGA of the seabed observation system controller and the observation sensors connected to FPGA do not work. The ARM processor works and sleeps periodically. The underwater acoustic communication module is continuously powered, and the seabed observation system communicates with the ship-borne operation monitoring system periodically. The pressure sensor does not work, and the altimeter and azimuth attitude sensor collect data periodically.

Seabed observation process: The seabed in-situ long-term observation sensors, altimeter, pressure sensor, and azimuth attitude sensor work periodically, and the non-operating state is powered down. The controller of the seabed observation system works in the working mode and the standby mode periodically. The underwater acoustic communication module is continuously powered, and the sea surface relay transmission buoy system, ship-borne remote monitoring system, and land-based remote monitoring system communicate with the seabed observation system periodically.

Seabed pull up process: The FPGA of the seabed observation system controller and the observation sensors connected to FPGA do not work. The ARM processor works and sleeps periodically. The underwater acoustic communication module is continuously powered, and the seabed observation system communicates with the ship-borne operation monitoring system periodically. The pressure sensor does not work, and the altimeter and azimuth attitude sensor collect data periodically.

The seabed observation system recovery process: The FPGA of the seabed observation system controller and the observation sensors connected to FPGA do not work. The ARM processor works and sleeps periodically. The underwater acoustic communication module is continuously powered, and the seabed observation system communicates with the ship-borne operation monitoring system periodically. The altimeter, pressure sensor, and azimuth attitude sensor connected with the ARM processor collect data periodically.

## 3. Key Design of SRSS/ILMO

### 3.1. System Anti-Crash Mechanism Design

Controller system crash is an inevitable problem in system reliability design. The particularity of the marine environment and the non-maintainability of an unattended system put forward higher requirements for the anti-crash performance of the seabed observation system. The anti-crash design of SRSS/ILMO adopts a two-stage anti-crash mechanism to respectively monitor the cold reset and hot reset of the system.

**Figure 8:**
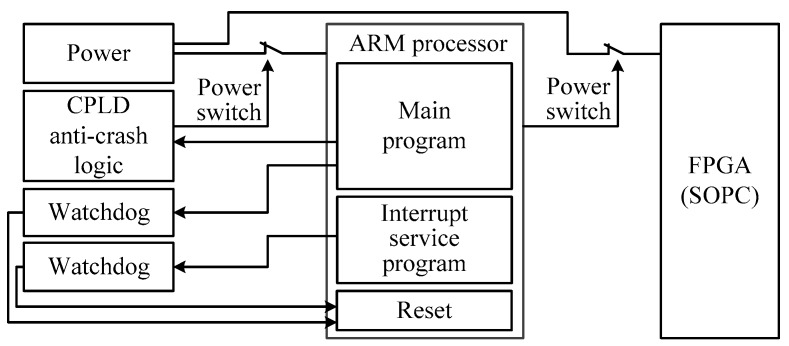
Two-stage anti-crash mechanism.

The system’s hot reset monitoring is a traditional watchdog design. When the program is disturbed and the program pointer jumps out of the active program area, the watchdog can effectively suppress the crash through hot reset. However, once the program enters an infinite loop containing the command of the feeding watchdog dog, the microcontroller will not be reset effectively. Therefore, SRSS/ILMO adopts a CPLD based cold reset anti-crash design. If the CPLD logic does not accept the normal monitoring signal, the control logic will perform cold reset processing; powering off the system for a certain period of time, and then re-powering the system. The two-stage anti-crash mechanism is shown in Figure 8.

### 3.2. Fault Detection and Automatic Isolation Design

**Figure 9:**
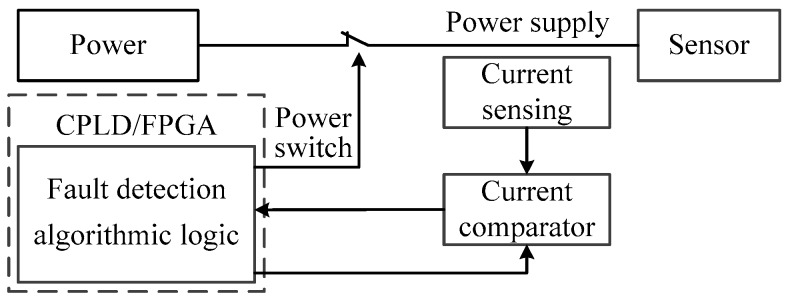
Fault detection and isolation design.

SRSS/ILMO adopts the overcurrent protection scheme consisting of digital processing logic and fast comparator to realize automatic fault isolation of external sensors or other devices. The fast comparator is used to monitor the power supply current according to the threshold set by the CPLD or FPGA. The digital processing logic integrates the current detection signal and gives the system fault judgment while guaranteeing the normal power-on process and power supply of the system. Once the system fault judgment is given, the digital processing logic will cut off the power supply of external equipment. The fault detection and isolation system diagram is shown in Figure 9.

### 3.3. Low Power Design

The seabed observation system of SRSS/ILMO works with limited power on the seabed. A low power consumption design is needed and its scheme is as follows.

**Figure 10:**
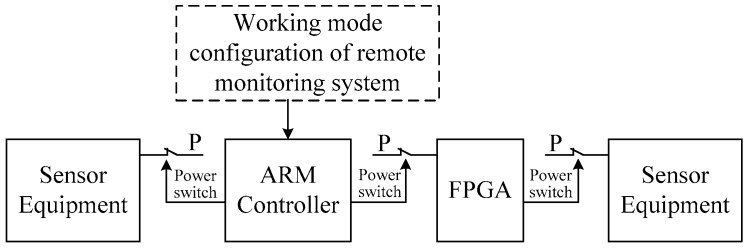
Hierarchical control of power supply.

First, the system adopts low-power chips such as the ARM processor. Second, on the basis of using low power devices, the system adopts a modular design method based on operation function. ARM connects with the underwater acoustic communication module, PA200, TCM XB, pressure sensor, and penetration system needed in the placement and recovery process, seabed penetration and pull up process. Seabed in-situ long-term observation sensors are connected to FPGA. Finally, based on the function module division, the method of hierarchical control of the power supply is adopted to reduce the power consumption of the system. As shown in Figure 10, the power supply of the sensor can be controlled by the FPGA according to the observation task, ARM can control the total power supply of the part of the FPGA according to the work task, and ARM can control the power supply of the external equipment connected to itself according to the work task.

The power consumption of the modules of the SRSS/ILMO, used in the project of seabed engineering geology in-situ long-term real-time surveying, is shown in Table 1. The minimum power consumption of the SRSS/ILMO is 0.96 W.

### 3.4. Communication Protocol Design

The Modbus protocol is a universal UART serial communication protocol for controllers. It is located in the application layer of the OSI model. The Modbus protocol can be used to connect different devices into an industrial network. Modbus has two transmission modes (ASCII and RTU). Referring to the RTU mode of Modbus protocol, the data frame format and detection domain of Modbus protocol is used to form the SRSS/ILMO communication protocol. It makes the system more secure and reliable in control and data transmission.

**Figure 11:**
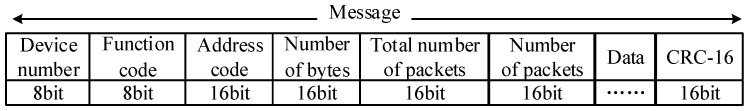
The data frame format of SRSS/ILMO.

As shown in Figure 11, the data frame format of SRSS/ILMO includes device number, function code, address code, number of bytes, total number of packets, number of packets, data, and CRC check. The device number is to distinguish the source of upload data and control command. The fixed device number is set to remote monitoring system, sea surface relay transmission buoy, and seabed observation system respectively. The function code is to distinguish whether the transmitted command is a sensor switch or a data command. The address code is to distinguish different sensors. To overcome sudden errors in underwater acoustic communication, data transmission adopts sub-packet transmission. In order to prevent sub-packet loss in data transmission, the total number of packets and number of packets are added in the data frame. CRC-16 check is used to ensure no data corruption during data transfer.

**Figure 12:**
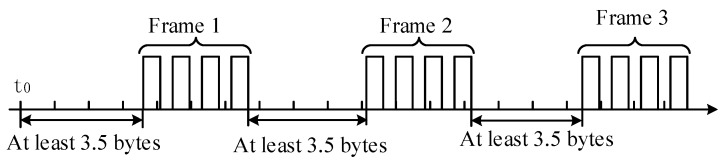
The 3.5 characters interval mechanism.

In Modbus protocol, the message is distinguished by a spatial interval of at least 3.5 characters. Using the interval time, the receiving device can judge whether the transmission ends. The 3.5 characters interval mechanism is shown in Figure 12. SRSS/ILMO uses this time interval method to design an UART serial communication program to improve the reliability of the UART serial interrupt service program.

## 4. Data Transmission Performance of SRSS/ILMO

As shown in Figure 1, the proposed SRSS/ILMO has two data transmission modes, including remote monitoring with satellite and underwater acoustic communication, and ship-borne monitoring with underwater acoustic communication. Based on the above application of submarine engineering geological observation, the performance of the two data transmission modes is analyzed. In the application, the Beidou satellite communication module and the AquaSeNt underwater acoustic communication module are used. The Beidou communication data rate is 77 bytes/min, and the underwater acoustic communication data rate is 2 kbps. The one-way underwater acoustic communication distance is 1500 m. Therefore, in the application of remote real-time observation including satellite communication, the data transmission bottleneck of the system is satellite communication. The data transmission performance of the system including Beidou satellite communication is shown in Table 2. When conducting parallel resistivity observation, the system can achieve the maximum data transmission capacity of 33,272 bytes per day. In ship-borne applications, the SRSS/ILMO can only use underwater acoustic communication to enhance the data transmission capability. Table 3 shows the data transmission performance only using underwater acoustic communication. For the bottleneck problem of the satellite communication mode, higher performance satellites can be adopted, such as the Iridium satellite and Beidou satellite with multiple cards. If the water surface relay platform is stable enough, directional satellite communication with higher performance can be adopted.

## 5. Test Results

### 5.1. System Reliability Verification

In order to verify the CPLD/FPGA based system design of the anti-crash mechanism, fault detection and automatic isolation mechanism, the simulation method based on Mentor’s Modelsim and the actual system test method based on oscilloscope monitoring are adopted respectively. Modelsim is the hardware description language (HDL) simulation software which supports the hybrid simulation of VHDL and Verilog HDL. It is the preferred simulation software for the design of CPLD/FPGA [19].

(1). System Anti-Crash Mechanism Verification

**Figure 13:**
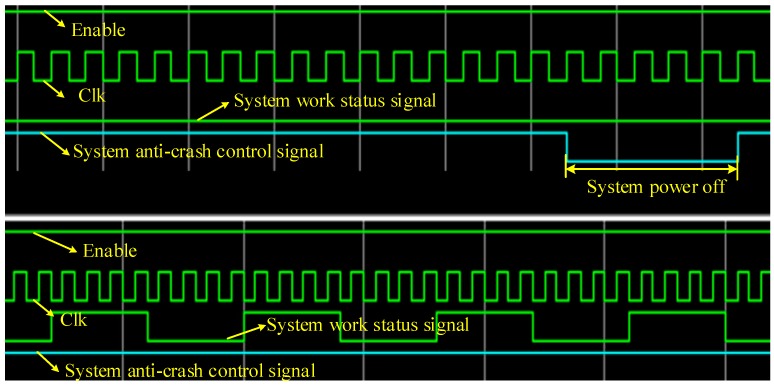
System anti-crash logic simulation results.

Figure 13 shows the Modelsim based simulation results of the system anti-crash logic design. The above timing diagram shows that the CPLD logic detected an abnormal system work status signal to give the system a power-off control signal. The below timing diagram shows that the CPLD logic did not give a power-off control signal because a normal signal was detected.

**Figure 14:**
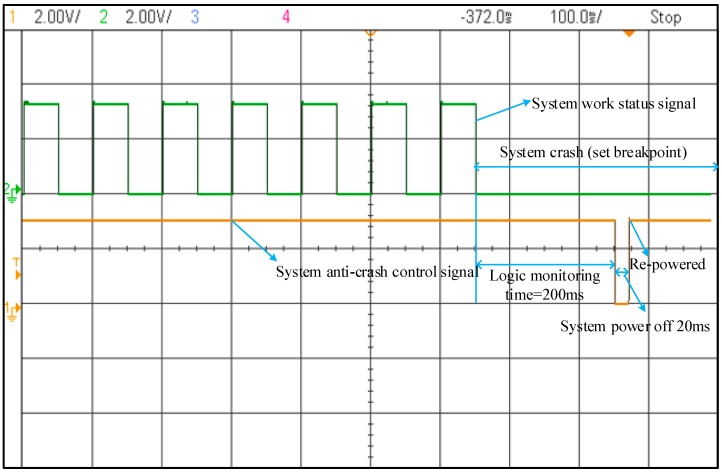
System anti-crash function test results.

In order to further verify the correctness of logical design. The system is debugged online. An oscilloscope is used to observe the system work status signal and the system anti-crash control signal. As shown in Figure 14, the green line is the system work status signal and the yellow line is the system anti-crash control signal. When the system crashes (executing to program breakpoint) for 200 ms, the system anti-crash control signal is given. The test results show that the system anti-crash mechanism is effective.

(2). Fault Detection and Automatic Isolation Verification

SRSS/ILMO adopts the overcurrent protection scheme consisting of digital processing logic and fast comparator to realize automatic overcurrent fault isolation of external sensors or other devices. The system external power supply circuit switch uses P-channel MOS IRF5210S which is the weakest part of the power supply circuit. Therefore, the fault detection and automatic isolation design is based on the safe operating conditions of IRF5210S. According to the datasheet, the safe operating condition of IRF5210S is|$I_{D}$| = 200 A @ duration ≤ 100 μs. Considering the safety margin and the working current of external sensors, the protection current threshold is set to 3 A, as shown in Table 4. In order to avoid the influence of peak current in the power-on process, CPLD digital logic adopts a time integration algorithm. The integration time is set to 20 μs.

**Figure 15:**
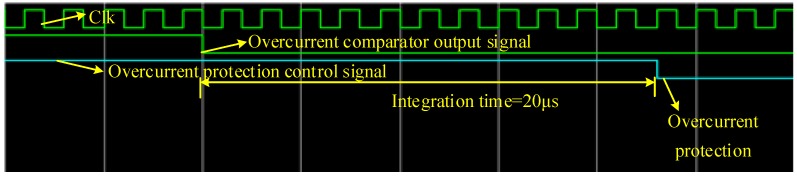
Fault detection and automatic isolation logic simulation results.

Figure 15 shows the Modelsim based simulation results of the system fault detection and automatic isolation design. When the overcurrent comparator output signal is low, the integrator starts working. When the integral value reaches the threshold, the logic gives an over-current protection signal.

**Figure 16:**
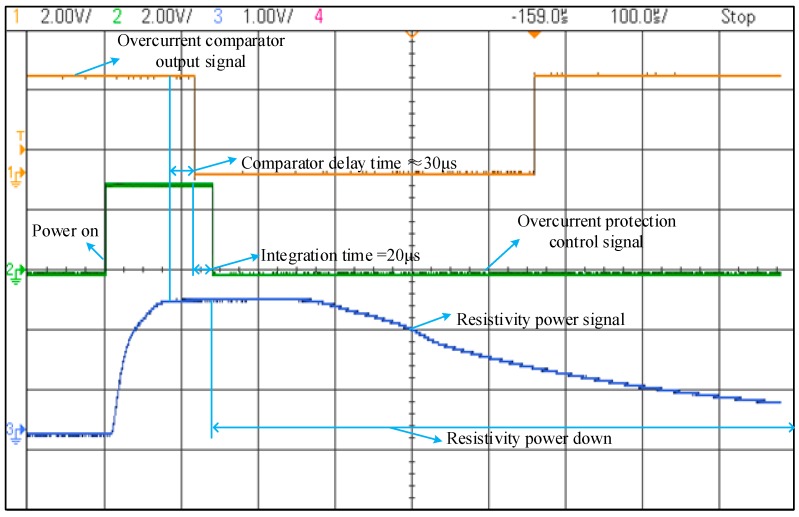
Fault detection and automatic isolation test results.

In order to further verify the correctness of logical design. The system is debugged online. An oscilloscope is used to observe the overcurrent comparator output signal, over-current protection control signal, and the external power supply signal. As shown in Figure 16, the yellow line is the overcurrent comparator output signal, the green line is the over-current protection control signal, and the blue line is the external power supply signal. The comparator circuit operation is delayed about 30 μs, and the integrator is set to an integration time of 20 μs, so it takes about 50 μs from fault detection to isolation, and the protection time is in the safe operation conditions. The test results show that the system fault detection and automatic isolation design is effective.

### 5.2. Harbor Environment Based System Test

**Figure 17:**
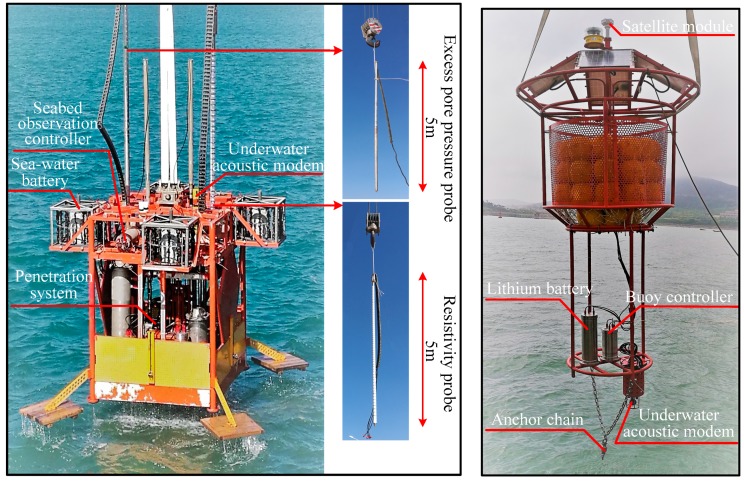
The physical photos of the SRSS/ILMO system.

In order to further verify the reliability of the system as a whole, the SRSS/ILMO used in seabed engineering geological observation was tested in the harbor environment at the National Deep Sea Center in Qingdao, China. The test site is a stable seabed at the near-shore, the geology is basically silt, and the water depth is about 15 m. The physical photos of the SRSS/ILMO system for testing are shown in Figure 17. Effective observation data of seawater battery, resistivity, and pore pressure were obtained. The system was subjected to several continuous observation experiments, and the longest continuous observation lasted for 35 days. The experiment results showed the feasibility and reliability of the proposed SRSS/ILMO.

(1). Sea-Water Battery Data Analysis

**Figure 18:**
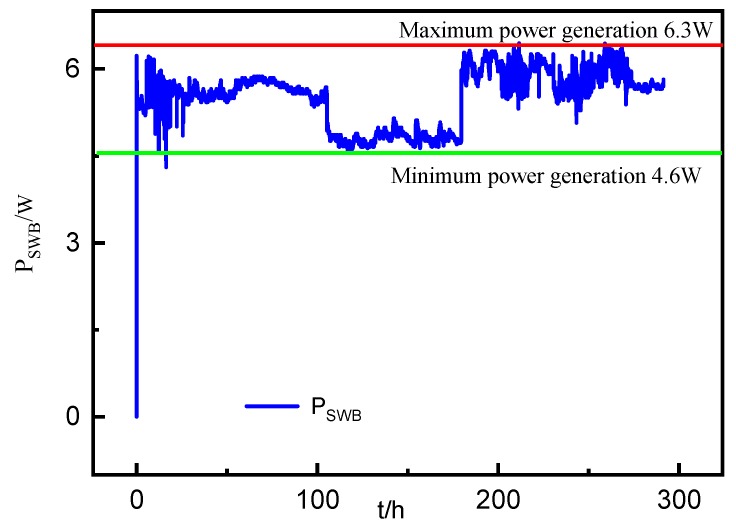
Power generation of the seawater battery.

The sea-water battery is the power supply module of the seabed observation system in the SRSS/ILMO used in the seabed engineering geological observation. As shown in Figure 18, the seawater battery is placed on the four ends of the seabed observation platform. Four seawater batteries with an electrochemical capacitor (SWB-EC) are used to charge the lithium batteries in the seabed observation platform.

Figure 18 shows the power generation of the seawater battery when working in the shallow water of the harbor environment test. The figure shows that the average power generation of the seawater battery is about 5.5 W, and the electric energy generation is 132 W per day. Throughout the test process, a complete and continuous monitoring for the seawater battery power generation process is presented by the SRSS/ILMO.

(2). Resistivity Data Analysis

**Figure 19:**
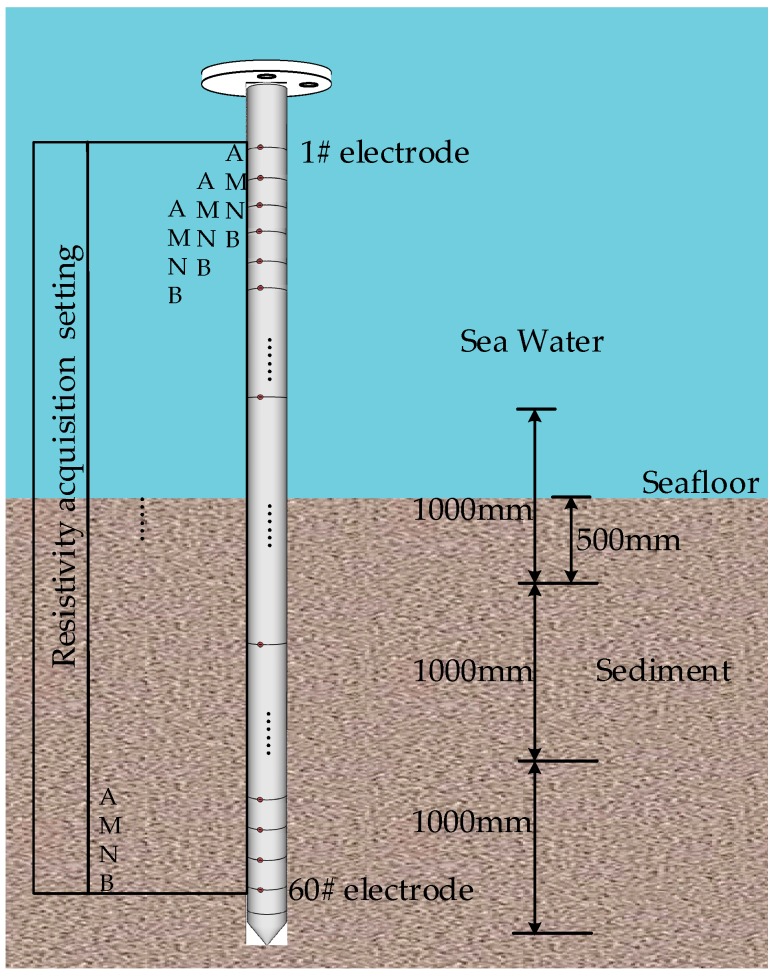
Diagram of resistivity probe.

The resistivity sensor is to detect a sediment depth of 3 m. The equipment platform is 2 m high. So a resistivity probe with 5 m height is used. The resistivity probe consists of 60 electrodes with an interval of 80 mm. Each electrode can be set by four switches as nodes A, B, M, or N. In the harbor test, the electrode set is A M N B polling from 1# to 60#, as shown in Figure 19. The probe penetrates into the seabed with a depth of about 2.5 m.

**Figure 20:**
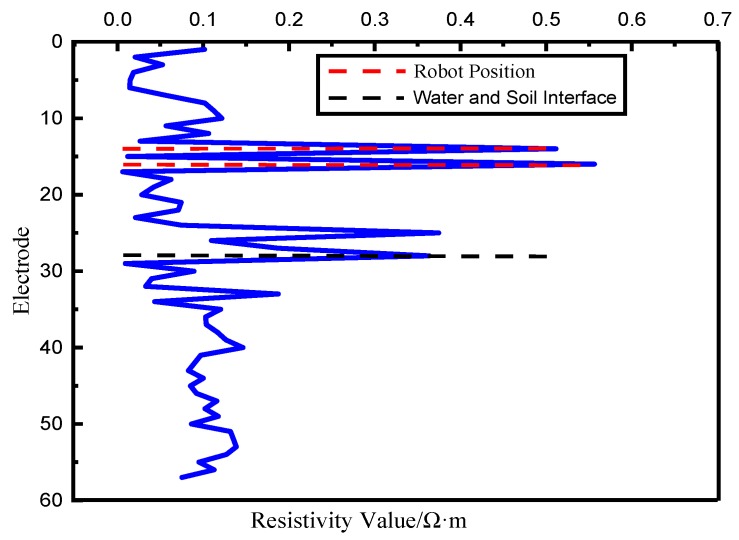
Single resistivity measurement results.

Figure 20 shows the single resistivity measurement results. It can be seen that the 14th electrode and the 16th electrode are affected by the manipulator mechanism. At the 28th electrode, where the interface position of seawater and sediment is situated, the resistivity value changes and jumps. It also can be seen that the greater the depth of the seafloor sediment, the greater the soil resistivity.

**Figure 21:**
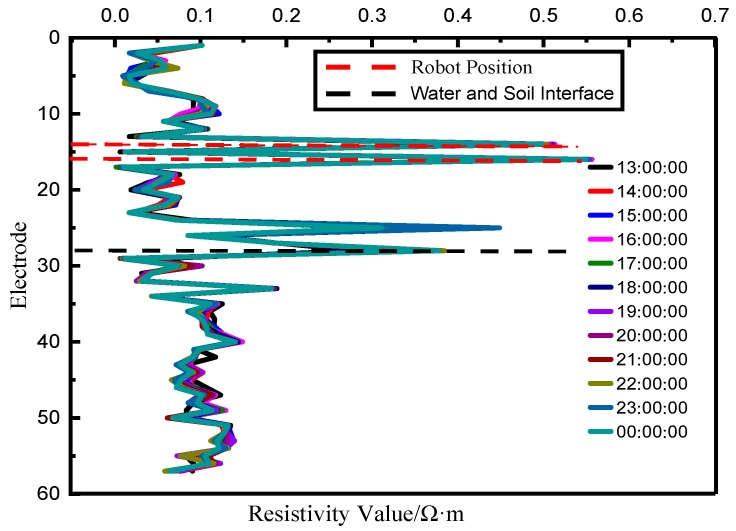
Continuous multiple resistivity measurement result.

As shown in Figure 21, the continuous resistivity collection was done every hour from 13:00:00 on 29 November to 00:00:00 on 30 November. It can be seen that the resistance values of the 60 electrodes did not change significantly for no obvious geology slippage in the harbor. The test result shows that the SRSS/ILMO is feasible and reliable, and there is no data packet loss during the test.

(3). FBG-PPS Data Analysis

**Figure 22:**
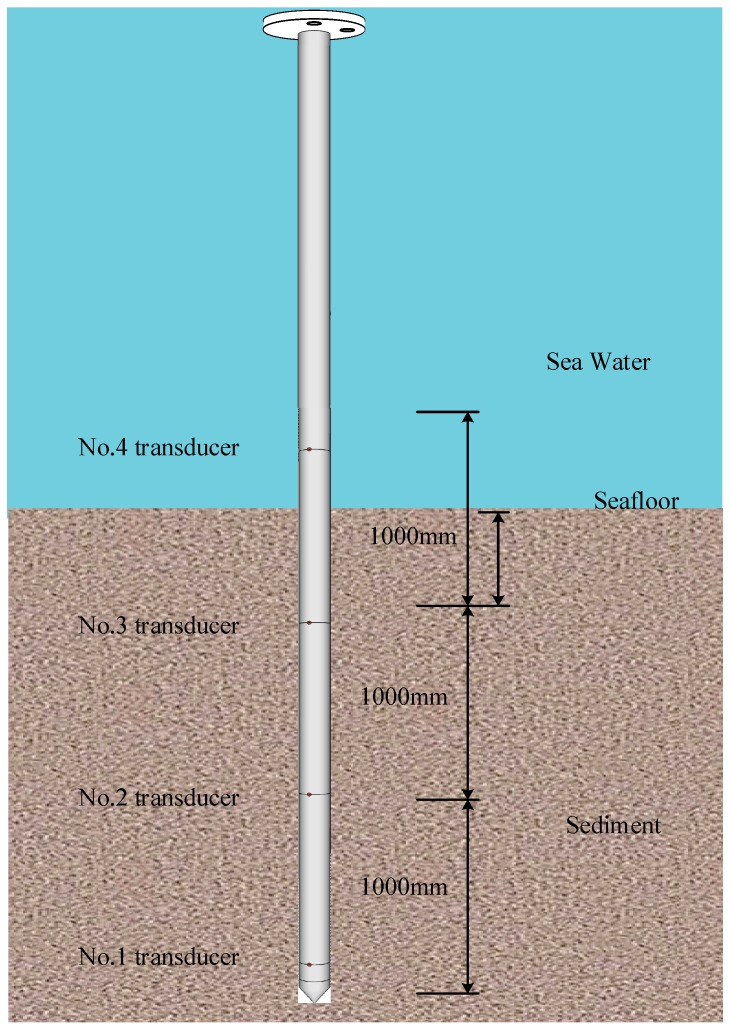
Diagram of the excess pore pressure probe.

The excess pore pressure probe is buried in the sediment to a depth of 2.5 m during the harbor test, as shown in Figure 22. It has three pressure sensors fully embedded in sediment and one exposed in seawater.

**Figure 23:**
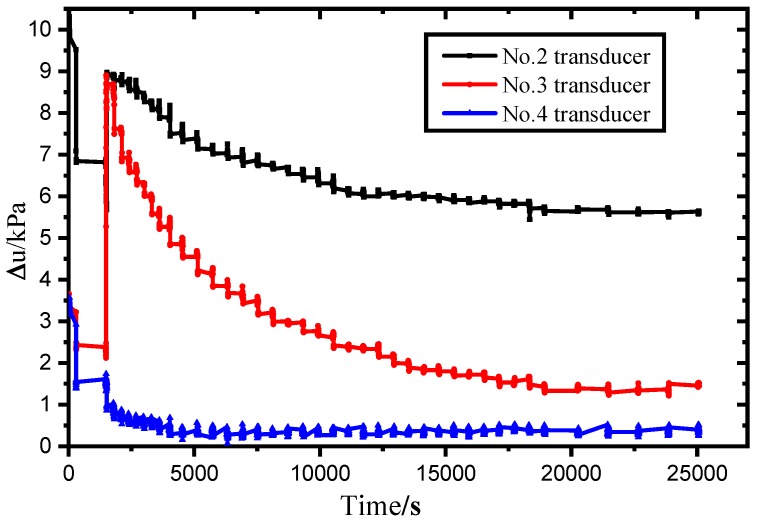
The excess pore pressure observation result.

As shown in Figure 23, the excess pore pressure caused by the penetration disturbance reaches the peak value, and the peak values of the perforation pressure of the 2# and 3# sensors are 8.97 kPa and 8.89 kPa respectively, and the 4# sensor’s peak fluctuation occurs with a peak of about 1.73 kPa. The 1# transducer failed during penetration and failed to return valid data.

**Figure 24:**
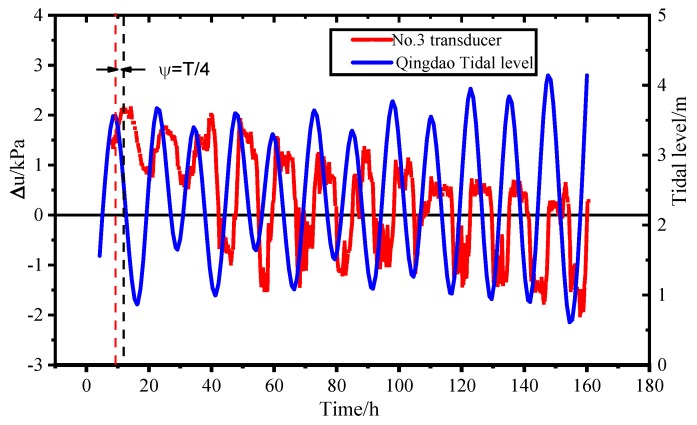
Pressure value compared with the tidal data.

Figure 24 is a comparison between the excess pore pressure observation values and local tidal data. The tidal data comes from the local meteorological release in Qingdao. As shown in Figure 24, the corresponding change trend relationship between the measured values of excess pore pressure and the local tides in Qingdao is similar, so the change of the super-porosity of the seafloor sediments in the harbor is mainly affected by hydrostatic and tidal effects [20]. The sensor is buried in the sedimentary layer. The pore pressure in the sedimentary layer is affected by the water environment (tidal etc.) on the surface of the seabed and the sedimentary environment (seepage, etc.), and the pore pressure changes in the sedimentary environment. The change in the water environment (tidal, etc.) relative to the surface of the seabed has characteristics such as time lag and amplitude attenuation. In the early stage, the deposition environment of the sensor is unstable and the measured pore pressure is relatively high. With the accumulation of observation time, the measured pore pressure is closer to the actual value, and the attenuation phenomenon is also more obvious, so the value is relatively small.

## 6. Related Works Comparison

Table 5 shows the comparison of the proposed SRSS/ILMO system with the other seabed observation systems. Comparisons are made in terms of self-contained storage, short-term real-time observation, long-term real-time observation, wire/wireless, networking, layout flexibility etc. Self-contained storage refers to whether the seabed observation system has its own data storage, short-term refers to the duration of less than one month (days or weeks), long-term refers to the duration of more than one month (months or years), real-time observation refers to whether it supports remote real-time transmission of observation data, wire/wireless refers to the communication mode of remote data transmission, networking refers to whether it supports the application of networking observation, and the layout flexibility refers to the operation convenience and the observation sites limitation. At present, with the development of low power and small volume storage technology, almost all kinds of submarine observation systems carry data memory to support self-contained storage. The in-situ self-contained observation system, the most commonly used ocean observation system at present, does not support the remote real-time transmission of observation data, and is easy to deploy without the limitation of observation sites. The ship-borne short-term observation system, which communicates with the mother ship in real-time through wired communication during the observation period, has real-time observation ability. It is convenient to deploy and is not restricted by observation sites. However, limited by the duration of mother ship operation (high operation cost), it does not support long-term observation. The sea-floor observation network, which is based on wired communication, does not depend on the mother ship and has the ability of long-term real-time observation. However, the wired networking leads to poor convenience of deployment, and the observation sites are severely limited. The subsurface buoy based real-time observation system, which adopts wireless communication above the water surface and wired communication below the water surface, does not depend on the mother ship and has the ability of long-term real-time observation. However, the underwater wired transmission leads to the poor convenience of deployment. The SRSS/ILMO system proposed in this paper adopts wireless communication above and below the water surface. The observation does not depend on the mother ship. It has the ability of long-term real-time observation. The underwater wireless transmission improves the convenience of system deployment. In summary, compared with the self-contained observation system, the SRSS/ILMO has the advantages of real-time observation; compared with the ship-borne real-time observation system, the SRSS/ILMO has the advantages of long-term in-situ observation; compared with the submarine cable network observation system and the subsurface buoy based real-time observation system, the SRSS/ILMO has the advantages of flexible deployment. 

## 7. Conclusions

Aiming at the real-time observation requirements in marine science and ocean engineering, based on underwater acoustic communication and satellite communication technology, a seabed real-time sensing system for in-situ long-term multi-parameter observation applications (SRSS/ILMO) is proposed in this paper. Based on the current underwater acoustic communication and satellite communication technology, the SRSS/ILMO has an acceptable data transmission capability. The system overall design, hardware composition design, key module design, and performance analysis are given. Based on the harbor environment, an application test of the submarine engineering geological observation was carried out. The preliminary test results show the effectiveness of the developed system. In the future, the deep-sea environment test of SRSS/ILMO will be carried out, and the system will be extended to other seabed in-situ long-term real-time multi-parameter observation applications.

## Figures and Tables

**Table 1:** Power consumption of each module of the seabed observation system.

Module	Power	Work Time	Average Power
Seabed observation controller (Work)	4.44 W	―	―
Seabed observation controller (Standby)	0.96 W	―	―
AquaSeNT Modem ^1^ (Transmit)	20 W	―	―
AquaSeNT Modem ^1^ (Receive)	0.7 W	―	―
AquaSeNT Modem ^1^ (Standby)	0.12 W	―	―
Resistivity	9 W	8 min/hour	1.2 W
FBG-PPS	15 W	4 min/hour	1 W
PA200	1.92 W	1 min/hour	0.032 W
TCM XB	0.1 W	1 min/hour	0.0017 W

^1^ Underwater acoustic communication module.

**Table 2:** Data transmission capability of SRSS/ILMO with the Beidou satellite.

Sensors	Bytes	Single Time	Frequency	Total Bytes
Resistivity serial acquisition	467	28 min	52/day	24,284
Resistivity parallel acquisition	4159	178 min	8/day	33,272
FBG-PPS	1034	44 min	32/day	33,088
Sea-water battery	70	4 min	360/day	25,200
PA200	9	2 min	720/day	6480
TCM XB	21	2 min	720/day	15,120

**Table 3:** Data transmission capability of SRSS/ILMO using underwater acoustic communication.

Sensors	Bytes	Single Time	Frequency	Total Bytes
Resistivity serial acquisition	467	435 s	198/day	92,466
Resistivity parallel acquisition	4159	610 s	141/day	586,419
FBG-PPS	1034	95 s	909/day	939,906
Sea-water battery	70	1 s	86,400/day	6,048,000
PA200	9	1 s	86,400/day	777,600
TCM XB	21	1 s	86,400/day	1,814,400

**Table 4:** Operating parameters of external sensors of SRSS/ILMO.

Sensors	Voltage	Normal Working Current	Protection Threshold Current
Resistivity	24 V	0.375 A	3 A
Modem	16 V	0.6 A	3 A
FBG-PPS	12 V	1.25 A	3 A
PA200	12 V	0.08 A	3 A
TCM XB	5 V	0.02 A	3 A

**Table 5:** Comparison between the proposed SRSS/ILMO and other systems.

System	Self-Contained Storage	Short-Term Real-Time Observation	Long-Term Real-Time Observation	Wire/Wireless	Networking	Layout Flexibility
SRSS/ILMO	yes	yes	yes	wireless	yes	high
Subsurface buoy based real-time observation system [21]	yes	yes	yes	wire/wireless	yes	medium
Sea-floor observation network [12,13,14,15,16,17,18]	yes	yes	yes	wire	yes	low
In-situ self-contained system [6,7,8]	yes	no	no	no	no	high
Shipborne short-term observation system [9,10,11]	yes	yes	no	wire	no	high
